# Maternal haemoglobin concentrations before and during pregnancy and stillbirth risk: a population-based case-control study

**DOI:** 10.1186/s12884-016-0924-x

**Published:** 2016-06-03

**Authors:** Siavash Maghsoudlou, Sven Cnattingius, Olof Stephansson, Mohsen Aarabi, Shahriar Semnani, Scott M. Montgomery, Shahram Bahmanyar

**Affiliations:** Clinical Epidemiology Unit, Department of Medicine Solna, Karolinska Institute, Karolinska Hospital, SE-171 76 Stockholm, Sweden; Faculty of Medicine, Golestan University of Medical Sciences, Gorgan, Iran; Division of Obstetrics and Gynecology, Department of Women’s and Children’s Health, Karolinska University Hospital and Institute, Stockholm, Sweden; Faculty of Medicine, Mazandaran University of Medical Sciences, Sari, Iran; Clinical Epidemiology and Biostatistics, School of Medical Sciences, Örebro University, Örebro, Sweden; Research Department of Epidemiology and Public Health, University College London, London, UK; Clinical Epidemiology Unit & Center for Pharmacoepidemiology, Department of Medicine, Karolinska Institute, Solna, Sweden

**Keywords:** Stillbirth, Maternal anaemia, Haemoglobin concentration, Haemoglobin dilution, Adverse pregnancy outcome

## Abstract

**Background:**

Results of previous studies on the association between maternal haemoglobin concentration during pregnancy and stillbirth risk are inconclusive. It is not clear if haemoglobin concentration before pregnancy has a role. Using prospectively collected information from pre-pregnancy and antenatal visits, we investigated associations of maternal haemoglobin concentrations before and during pregnancy and haemoglobin dilution with stillbirth risk.

**Methods:**

In a population-based case–control study from rural Golestan, a province in northern Iran, we identified 495 stillbirths (cases) and randomly selected 2,888 control live births among antenatal health-care visits between 2007 and 2009. Using logistic regression, we estimated associations of maternal haemoglobin concentrations, haemoglobin dilution at different stages of pregnancy, with stillbirth risk.

**Results:**

Compared with normal maternal haemoglobin concentration (110–120 g/l) at the end of the second trimester, high maternal haemoglobin concentration (≥140 g/l) was associated with a more than two-fold increased stillbirth risk (OR = 2.31, 95 % CI [1.30–4.10]), while low maternal haemoglobin concentration (<110 g/l) was associated with a 37 % reduction in stillbirth risk. Haemoglobin concentration before pregnancy was not associated with stillbirth risk. Decreased haemoglobin concentration, as measured during pregnancy (OR = 0.61, 95 % CI [0.46, 0.80]), or only during the second trimester (OR = 0.75, 95 % CI [0.62, 0.90]), were associated with reduced stillbirth risk. The associations were essentially similar for preterm and term stillbirths.

**Conclusions:**

Haemoglobin concentration before pregnancy is not associated with stillbirth risk. High haemoglobin level and absence of haemoglobin dilution during pregnancy could be considered as indicators of a high-risk pregnancy.

**Electronic supplementary material:**

The online version of this article (doi:10.1186/s12884-016-0924-x) contains supplementary material, which is available to authorized users.

## Background

Results of previous studies on associations between maternal haemoglobin concentration and risks of preterm birth, low birth weight, small for gestational age, and stillbirth are inconclusive [1, 2]. We and others have previously reported that both low and high haemoglobin concentration during pregnancy may increase stillbirth risk [2–4]. Yet, other studies have found that increased stillbirth risk is restricted to women with either low [5] or high [6] haemoglobin values.

During pregnancy, there is significant change in maternal haemostatic profile [7], including haemoglobin dilution due to the expanding serum component of the blood [8]. Disruption in this process, possibly due to pathology in early pregnancy [9], seems to be associated with adverse pregnancy outcomes [2]. However, it is not clear if haemoglobin concentration before pregnancy modifies this association.

In the Golestan province in northern Iran, the primary health care organization provides modern antenatal and obstetric care to virtually all pregnant women. In the rural part of the province, most pregnancies are planned and most women have, following the recommendations of the Alma Ata conference, a pre-pregnancy visit followed by several antenatal visits [10]. Information on maternal haemoglobin concentrations measured before pregnancy and repeatedly during pregnancy is routinely recorded.

In this population-based case–control study of stillbirth from the rural area of the Golestan province in northern Iran, we studied associations of haemoglobin concentrations before and during pregnancy with stillbirth risk, and also studied associations of maternal haemoglobin dilution (average weekly change in haemoglobin concentration) during the first and second trimester of pregnancy with stillbirth risk.

## Methods

### Study setting

The study was performed in rural areas of the Golestan province, in northeast Iran. Golestan province has a population of approximately 1,700,000 inhabitants (50 % living in rural areas), and has approximately 17,000 births annually. The public health care system in Iran specifies that each rural health house is responsible for providing care and recording information before and during pregnancy and after delivery. Blood profile and other laboratory data are routinely assessed at the pre-pregnancy visit and twice during pregnancy. Such information is prospectively recorded in the family health files held by approximately 600 rural health houses.

### Study design

The study design has been described in full elsewhere [11]. All identified singleton stillbirths in the rural areas of Golestan Province between 2007 and 2009 were selected as cases. Stillbirth was defined as delivery of a baby without any vital signs at 28 weeks of gestation or later. We identified 501 singleton stillbirths, and after excluding stillbirths without any maternal blood haemoglobin concentration records during pregnancy, 495 stillbirths were selected as cases. Controls were selected using a stratified random sampling method. As growth rate vary between regions of Golestan province, we defined each region as a stratum and calculated the sample size for each stratum. The proportion of registered pregnancies selected was based on the population growth rate of the region. Each pregnancy had the same probability to be selected. We selected controls using random digits generated by computer. We aimed to have at least five controls per case after excluding births before 28 gestational weeks, multiple births, and stillbirths. The control group comprised 2,888 live singleton births with a gestational age of at least 28 completed weeks.

Information on maternal and pregnancy characteristics were collected from medical records by midwifes working at the health centres. Data were abstracted from pre-pregnancy, pregnancy and delivery records, and information was entered using Access software by ten specially trained medical students. Each record, including information on the mother, children and spouse, received a unique code at the time of data collection and these codes were used during data entry to identify individuals and make it possible to analyse the data anonymously. The measurement of socioeconomic circumstances was based on husband’s profession (unskilled manual worker, skilled manual worker, self-employed, farmer, other occupations, and unemployed). Self-reported information on current or previous smoking and opium use was also collected. As a quality control, we collected and computerized 10 % of the data a second time. The variables that had more than 5 % mismatches were recollected and the data re-entered for all study subjects. If the data for a health centre had more than 5 % mismatches, all data were recollected and re-entered for that centre.

Based on the “Iranian National Antenatal Care” protocols in the primary health care system, laboratory testing (included complete blood count test, blood biochemistry profile, and urine analysis) was performed twice during pregnancy: at 6 to 10 and at 26 to 30 gestational weeks.

The mean gestational ages for the first and second haemoglobin concentration measurements were 9 weeks and 28 weeks for both cases and controls. *Haemoglobin dilution during the second trimester of pregnancy* was calculated as the difference between blood haemoglobin concentration at the first and last measurement during pregnancy, divided by the time period (completed weeks) between the two measurements (mean change in haemoglobin concentration per gestational week). Observations with a time interval of less than three weeks between two haemoglobin measurements were excluded from the analysis of haemoglobin dilution (*n* = 3).

Among all cases (*n* = 495) and controls (*n* = 2,888), 158 cases and 1,225 controls also had a recorded blood haemoglobin concentration during the year before pregnancy. For this group we also estimated *haemoglobin dilution during pregnancy,* defined as the difference between the pre-pregnancy and the last pregnancy measurement of blood haemoglobin concentration, divided by gestational age (in completed weeks) at the last measurement.

Gestational age was based on the time interval between date of delivery and date of the first day of the last menstrual period. Preterm delivery was defined as a delivery before 37 completed weeks. As a proxy measure for foetal growth restriction, we used small for gestational age (SGA), which was defined as a birth weight below the 10^th^ percentile (in the control group) for gestational age (week) and sex. Because of the limited number of preterm births in the control group, we could not estimate appropriate cut off limits for SGA in preterm deliveries. Maternal blood pressure was measured before pregnancy, at least 8 times during pregnancy and after delivery. Hypertension was defined as a measured systolic blood pressure ≥140 and/or diastolic blood pressure ≥90 during each trimester of pregnancy.

The study was approved by the Research Ethics Committee of Golestan University of Medical Sciences and the Karolinska Institutet regional ethics committee. The ethical committees did not require written informed consent for participation in the study.

### Statistical analysis

We used univariable and multivariate logistic regression models to estimate odds ratios (ORs) and 95 % confidence intervals (CIs) for the associations of maternal haemoglobin concentrations, haemoglobin dilution, with stillbirth risk. As the outcome is rare in both exposed and unexposed mothers, the odds ratio approximates the risk ratio and the results are described in terms of risk ratio throughout the paper. The multivariate models were adjusted for maternal age, pre-pregnancy body mass index (BMI), height, gestational age at the first haemoglobin measurement, parity (0, 1–2, >2), smoking status, region of residence, and husband’s profession. We also investigated possible nonlinear effects of age and BMI by introducing age squared and BMI squared into the adjusted model. However, these variables had not notable effects and were therefore not included in the final models.

Pregnancy-induced hypertensive diseases are related to both stillbirth risk [12] and haemoglobin concentration during pregnancy [13]. Information on preeclampsia or eclampsia was not available, but we performed a sensitivity analysis after excluding all subjects with a record of hypertension during pregnancy.

We estimated odds ratios for stillbirth associated with maternal haemoglobin concentrations before pregnancy, in the first trimester, and in the second trimester of pregnancy. We estimated the risks using both maternal haemoglobin concentration as a continuous variable (gram per litter [g/l]), and as a four-category measure (<110, 110–120, 120–139, and ≥140 g/l). We also estimated odds ratios for stillbirth associated with haemoglobin dilution in each gestational week: a) during pregnancy (>0.78, 0.78–0.01, and ≤ 0.00 g/l), and b) during the second trimester of pregnancy (>0.78, 0.78–0.01, and ≤ 0.00 g/l). The haemoglobin categories were based on tertiles of the distribution in the control group.

A multiple imputation method was used to provide data where there were missing values for maternal age (32 among controls) and husband’s profession (16 among cases and 79 among controls) [14]. The MI procedure (SAS software) with five imputations was used. We also performed a sensitivity analysis by restricting the analysis to observations without any missing data.

SAS software version 9.3 was used for analysing the data.

## Results

Maternal characteristics of cases and controls are presented in the Table 1. Associations of the maternal haemoglobin concentrations with risk of stillbirth are presented in Table 2. Maternal haemoglobin concentration measured before pregnancy was not associated with stillbirth risk. Compared with normal maternal haemoglobin values (110–120 g/l) at the end of the first trimester, high haemoglobin values (≥140 g/l) were associated with a 36 % increased risk of stillbirth. Compared with normal haemoglobin values at the second trimester of pregnancy, high maternal haemoglobin values were associated with a more than two-fold increased risk of stillbirth, and low haemoglobin concentration (≤110 g/l) was associated with a reduced stillbirth risk. We also estimated associations between the maternal haemoglobin concentrations and risk of stillbirth stratified by gestational age (preterm and term stillbirth), which showed no notable differences in the results (Additional file 1).

**Table 1:** Maternal characteristics for 495 stillbirths and 2,888 live singleton births in Golestan, Iran

	Cases	Controls
	n (%)	n (%)
Mother’s age, year		
≤ 19	72 (14.5)	363(12.6)
20-24	127 (25.7)	865 (30.0)
25–29	138 (27.9)	760 (26.3)
30–34	92 (18.6)	589 (20.4)
≥ 35	66 (13.3)	279 (9.7)
Missing	0 (0)	32 (1.1)
Body mass index		
< 18.5	39 (7.9)	200 (6.9)
18.5 to <25	180 (36.4)	1,426 (49.4)
25 to <30	117 (23.6)	655 (22.7)
30 to <35	56 (11.3)	230 (8.0)
≥ 35	26 (5.3)	60 (2.1)
Missing	77 (15.6)	317 (11.0)
Mother’s height, cm		
< 150	48 (9.7)	192 (6.6)
150–154	116 (23.4)	590 (20.4)
155–159	152 (30.7)	921 (31.9)
160–164	55 (11.1)	479 (16.6)
≥ 165	47 (9.5)	389 (13.5)
Missing	77 (15.6)	317 (11.0)
Parity		
0	256 (51.7)	1,140 (39.5)
1–2	192 (38.8)	1,338 (46.3)
≥ 3	32 (6.5)	189 (6.5)
Missing	15 (3.0)	221 (7.7)
Pregnancy induced hypertension		
	459 (92.7)	2,786 (96.5)
	36 (7.3)	102 (3.5)
Smoking		
No	488 (98.6)	2,803 (97.1)
Yes	7 (1.4)	85 (2.9)
Husband’s profession		
Unemployed	16 (3.2)	76 (2.6)
Non skill worker	206 (41.6)	1,318 (45.6)
Skill worker	55 (11.1)	275 (9.5)
Self-employed	78 (15.8)	327 (11.3)
Farmer	79 (16.0)	547 (18.9)
Other	44 (8.9)	266 (9.2)
Missing	17 (3.4)	79 (2.7)
Haemoglobin concentration before pregnancy (g/l)		
Mean (SD)	126.3 (10.4)	125.7 (11.4)
Median (Range)	130.0 (100–150)	120.0 (80–160)
Haemoglobin concentration at first trimester (g/l)		
Mean (SD)	124.5 (12.3)	122.7 (11.7)
Median (Range)	120.0 (80 to 170)	120.0 (70 to 160)
Haemoglobin concentration at end of second trimester (g/l)		
Mean (SD)	117.1 (11.6)	113.5 (11.3)
Median (Range)	120.0 (80–150)	110.0 (70–170)
Region		
Aghghala	51 (10.3)	377 (13.1)
Aliabad	28 (5.7)	183 (6.3)
Azadshahr	32 (6.5)	142 (4.9)
Bandargaz	7 (1.4)	114 (3.9)
Galikesh	23 (4.6)	115 (4.0)
Gomishan	37 (7.5)	104 (3.6)
Gonbad	130 (26.3)	524 (18.1)
Gorgan	15 (3.0)	284 (9.8)
Kalaleh	65 (13.1)	361 (12.5)
Kordkoy	14 (2.8)	84 (2.9)
Maraveh	37 (7.5)	168 (5.8)
Minoodasht	23 (4.6)	235 (8.1)
Ramian	17 (3.4)	102 (3.5)
Torkaman	16 (3.2)	95 (3.3)

**Table 2:** Haemoglobin concentration and the risk of stillbirth

	Cases	Controls	Odds Ratio (95 % Confidence Interval)	
	n (%)	n (%)	Crude	Adjusted^a^
Haemoglobin concentration before pregnancy (g/l)				
< 110	4 (2.5)	31 (2.5)	1.04 (0.36–3.04)	1.23 (0.39–3.84)
110–120	72 (45.6)	582 (47.5)	Reference	Reference
121–139	49 (31.0)	360 (29.4)	1.10 (0.75–1.61)	0.97 (0.64–1.47)
≥ 140	33 (20.9)	252 (21.0)	1.06 (0.68–1.64)	0.83 (0.52–1.34)
Continuous	158 (100)	1,225 (100)	1.00 (0.99–1.02)	1.00 (0.98–1.01)
Haemoglobin concentration at first trimester (g/l) (gestational age weeks between 6 to 10)				
< 110	26 (5.3)	181 (6.5)	0.91 (0.59–1.40)	0.87 (0.55–1.37)
110–120	223 (46.1)	1,411 (50.5)	Reference	Reference
121–139	152 (31.4)	834 (30.0)	1.45 (0.92–1.44)	1.18 (0.94–1.49)
≥ 140	83 (17.1)	366 (13.1)	1.43 (1.09–1.89)	1.36 (1.01–1.81)
Continuous	484 (100)	2,792 (100)	1.01 (1.00–1.02)	1.01 (1.00–1.02)
Haemoglobin concentration at end of second trimester(g/l) (gestational age weeks between 26 to 30)				
< 110	37 (14.0)	523 (21.2)	0.64 (0.44–0.92)	0.63 (0.43–0.92)
110–120	180 (65.2)	1,618 (65.6)	Reference	Reference
121–139	40 (14.4)	268 (10.9)	1.34 (0.93–1.93)	1.19 (0.81–1.74)
≥ 140	18 (6.4)	59 (2.4)	2.74 (1.58–4.75)	2.31 (1.30–4.10)
Continuous	275 (100)	2,468 (100)	1.03 (1.02–1.04)	1.03 (1.01–1.04)

^a^Adjusted for maternal age, maternal height, maternal BMI, parity, smoking status, husband’s profession, region and gestational age at haemoglobin measurement

The associations between level of haemoglobin dilution and stillbirth risk are shown in Table 3. Compared with mothers who did not have any change in haemoglobin concentration during the second trimester, those with decreased haemoglobin concentration had a reduced stillbirth risk. For the subsample where information on pre-pregnancy haemoglobin concentration was available, we also estimated haemoglobin dilution during pregnancy (from before pregnancy to last measurement during pregnancy). Compared with those who did not have any change in haemoglobin concentration during pregnancy, those with decreased haemoglobin concentration had a reduced stillbirth risk. There was a dose–response association between haemoglobin dilution as an exposure and stillbirth risk, both in the analysis of the second trimester and in the analysis of pregnancy.

**Table 3:** Haemoglobin dilution and the risk of stillbirth

	Cases	Controls	Odds Ratio (95 % Confidence Interval)	
	n (%)	n (%)	Crude	Adjusted^a^
Haemoglobin dilution during second trimester of pregnancy (g/l)				
> 0.78	70 (26.7)	765 (33.8)	0.57 (0.42–0.78)	0.54 (0.39–0.74)
0.78–0.01	73 (27.9)	750 (33.2)	0.61 (0.45–0.83)	0.62 (0.45–0.85)
0.00 ≤	119 (45.4)	745 (33.0)	Reference	Reference
P for trend				<0.0001
Continuous	262 (100)	2,260 (100)	0.76 (0.63–0.92)	0.75 (0.62–0.90)
Haemoglobin dilution during pregnancy (g/l)				
> 0.69	31 (20.0)	392 (33.1)	0.33 (0.21–0.51)	0.33 (0.21–0.52)
0.69–0.01	38 (24.5)	430 (36.3)	0.37 (0.25–0.56)	0.42 (0.27–0.66)
0.00 ≤	86 (55.5)	361 (30.5)	Reference	Reference
P for trend				<0.0001
Continuous	155 (100)	1,183 (100)	0.58 (0.45–0.74)	0.61 (0.46–0.80)

^a^Adjusted for maternal age, maternal height, maternal BMI, parity, gestational age at first haemoglobin measurement, smoking status, husband’s profession, and region

We also performed a sensitivity analysis after excluding all pregnancies with a record of hypertension (systolic blood pressure ≥140 and/or diastolic blood pressure ≥90) during pregnancy. After excluding 36 cases and 102 controls with hypertensive disorders, the results were virtually unchanged (data not shown).

Table 4 shows associations between average weekly change in haemoglobin concentration and risks of preterm and term stillbirth. There was no substantial difference between risks for term and preterm stillbirth using the categorical variable: higher levels of haemoglobin dilution were associated with lower risk of preterm stillbirth and there was a dose response association (p for trend <0.01). For term stillbirth, we observed a statistically significant 25 % risk reduction for an average of one g/l weekly change in haemoglobin during the second trimester. For preterm stillbirth, the corresponding point estimate was identical, but not statistically significant (Table 4).

**Table 4:** Haemoglobin dilution and risks of preterm and term stillbirth

	Preterm stillbirth			Term stillbirth		
	Cases (*n* = 81)	Controls (*n* = 2,260)	Odds ratio^a^ (95 % CI)^b^	Cases (*n* = 181)	Controls (*n* = 2,105)	Odds ratio^a^ (95 % CI)^b^
	n (%)	n (%)	n (%)	n (%)	n (%)	
Haemoglobin dilution during second trimester of pregnancy (g/l)						
> 0.78	22 (27.6)	765 (33.8)	0.49 (0.28–0.84)	48 (26.5)	716 (34.0)	0.55 (0.38–0.81)
0.78–0.01	20 (24.7)	750 (33.2)	0.50 (0.28–0.87)	53 (29.3)	701 (33.3)	0.67 (0.46–0.97)
0.00 ≤	39 (48.1)	745 (33.0)	Reference	80 (44.2)	688 (32.7)	Reference
P for trend			<0.01			<0.01
Continuous	81 (100)	2,260 (100)	0.75 (0.54–1.06)	181 (100)	2,105 (100)	0.75 (0.61–0.93)

^a^Adjusted for: maternal age, maternal height, maternal BMI, parity, gestational age at first haemoglobin measurement, history of smoking, husband’s profession, and region

^b^OR denotes Odds ratios and CI denotes confidence intervals

Table 5 shows associations between average weekly change in haemoglobin concentration and stillbirth stratified by SGA stillbirths and non-SGA stillbirths in term deliveries. Using the measure of change in haemoglobin concentration as a *continuous* variable, we observed a 40 % risk reduction of SGA stillbirth for each g/l weekly change in haemoglobin during the second trimester. Haemoglobin dilution was not associated with a reduced risk of non-SGA term stillbirth. Restricting analyses to observations without any missing data did not change the results notably.

**Table 5:** Haemoglobin dilution and risks of SGA and Non-SGA in term stillbirth

	SGA stillbirth			Non-SGA stillbirth		
	Cases (*n* = 55)	Controls (*n* = 215)	Odds ratio^a^ (95 % CI)^b^	Cases (*n* = 75)	Controls (*n* = 1,858)	Odds ratio^a^ (95 % CI)^b^
	n (%)	n (%)		n (%)	n (%)	
Haemoglobin dilution during second trimester of pregnancy (g/l)						
> 0.78	12 (26.4)	66 (34.0)	0.48 (0.19–1.22)	20 (26.7)	639 (34.4)	0.62 (0.34–1.11)
0.78–0.01	18 (25.5)	67 (33.3)	1.01 (0.43–2.39)	26 (34.7)	621 (33.4)	0.87 (0.50–1.52)
0.00 ≤	25 (48.1)	82 (32.6)	Reference	29 (38.7)	598 (32.2)	Reference
Continuous	55 (100)	215 (100)	0.59 (0.36–0.97)	75 (100)	1,858 (100)	0.95 (0.68–1.33)

^a^Adjusted for: maternal age, maternal height, maternal BMI, parity, gestational age at first haemoglobin measurement, history of smoking, husband’s profession, and region

^b^OR denotes Odds ratios and CI denotes confidence intervals

## Discussion

This population-based case–control study showed that haemoglobin concentration before pregnancy was not associated with stillbirth risk. High and low haemoglobin concentrations at the end of the second trimester were associated with an increased and a reduced stillbirth risk, respectively. Decreased haemoglobin concentration, during the entire pregnancy or during the second trimester, was also associated with reduced stillbirth risk.

Our findings are consistent with other studies, reporting positive associations between increasing maternal haemoglobin levels in early [2, 15] or late pregnancy [16] and risk of stillbirth or other adverse outcomes [13, 17–19]. During pregnancy, plasma renin activity increases [9, 20] and the atrial natriuretic peptide levels decrease to meet demands of new vascular beds [21, 22]. High maternal erythropoietin secretion during pregnancy causes an increase in red blood cell mass. However, this increase is less than the change in the plasma volume, which causes a 10 to 20 gram decrease in haemoglobin concentration per litre plasma at the end of the second trimester in normal pregnancy [7]. High blood haemoglobin levels may be a consequence of disturbance in the physiology of expanding the serum component of the maternal blood during pregnancy [23, 24].

We also observed that decreased haemoglobin concentration during pregnancy was associated with lower risk of stillbirth, regardless of initial haemoglobin level or time of the change in haemoglobin concentration. This protective effect was also observed in previous studies on stillbirth [2], preeclampsia [25], and low birth weight [25, 26]. However, previous studies did not have pre-pregnancy data to investigate the possible role of haemoglobin concentration before pregnancy. The association was essentially similar between preterm and term stillbirths, and if anything, more marked among SGA stillbirths in term pregnancies. These findings, which are consistent with the results of a Swedish study [2], may indicate that the risk of stillbirth related to high haemoglobin concentration during pregnancy might be associated with impaired foetal growth. We did not have enough data to investigate risks stillbirth risks associated with impaired foetal growth in preterm pregnancies or stillbirths with malformations.

Preeclampsia is associated with both stillbirth risk [12] and haemoglobin concentration during pregnancy [13] and this disorder could be an intermediate factor. As information on preeclampsia or eclampsia was not available, we reanalysed the data after excluding all subjects with a record of hypertension during pregnancy. We observed no notable change in the results. This is in contrast with the results of a Swedish study [2] in which the stillbirth-related risks associated with a high haemoglobin concentration increased when women with preeclampsia or eclampsia were excluded.

To the best of our knowledge, there are no published data on the association between haemoglobin concentration prior to pregnancy and risk of stillbirth. The lack of association between haemoglobin concentration before pregnancy and stillbirth in this study could be because women with a low haemoglobin concentration receive pharmacological treatment before pregnancy. Based on routines in the primary health care system in Iran, more than 90 % of pregnant women receive iron and other micro-supplements after the 16th week of pregnancy. Moreover, women of reproductive age undergo screening to identify and treat iron deficiency anaemia, particularly before pregnancy [10] which can increase haemoglobin concentration [27]. This practise eliminates to a large extent the possible effects of iron deficiency in our study. Iron deficiency can be a major contributory factor to both haemoglobin dilution and serious maternal anaemia, [7] which might increase risk of adverse pregnancy outcomes such as preterm birth and low birth weight [1]. A recent meta-analysis showed that prenatal iron use is associated with a significant increase in birth weight and reduction in risk of low birth weight [28]. However, prenatal daily iron supplement intake might also be associated with risk of higher haemoglobin concentration during pregnancy [29]. Therefore, it is questionable whether women with normal blood values benefit from taking iron supplements [30].

Strengths of the study include the use of prospectively collected information from antenatal visits. We included all cases and randomly selected controls with information on maternal blood profile, and extracted prospectively recorded information from family health records, which minimizes risks of selection and recall bias. This study was conducted in rural areas of Golestan province, where most pregnancies are planned, and some 97 % of pregnant women are in contact with the primary health care system.

This study has also some potential limitations. Maternal blood profile was tested in different laboratories which may had different methods and accuracy. As recorded haemoglobin concentrations were rounded to the nearest half unit, we did not have information on exact values. The number of women with a very low haemoglobin concentration was small and we could not estimate the risk of stillbirth among this group. As stillbirth was defined as delivery of a baby without any vital signs at 28 weeks of gestation or later, we did not have any information on very preterm stillbirth (less 28 weeks gestational age). This helps to explain the low proportion of preterm stillbirths in our study. Smoking is culturally not accepted in rural area of Iran, and some women may have not have reported history of smoking accurately. As we used prospectively collected information before and during pregnancy, possible misclassification of smoking status would be non-differential, and if anything, shift the association toward the null. Opium use exists among women of childbearing age in Iran [31]. In Golestan province most of the married women (more than 90 %) are housewives and we did not have any information about educational level of the mothers. Another limitation is that the husband’s profession, which is used as measure of socioeconomic conditions for families, may not be sufficiently discriminatory. We restricted our study to pregnancies in rural areas of the Golestan province and there may be concern regarding generalizability of the results. Exclusion of those with missing haemoglobin values might cause selection bias. Finally, we did not have any data to determine the cause of the stillbirth, specifically genetic testing, autopsy, or placental pathology or congenital defects; therefore we could not investigate difference in the association between haemoglobin concentration and stillbirth by malformation.

## Conclusions

In conclusion, this study showed that haemoglobin concentration before pregnancy was not associated with stillbirth risk. This study also provides evidence that elevated maternal haemoglobin level during pregnancy is a risk factor for stillbirth, and low haemoglobin level is inversely associated with stillbirth. High haemoglobin level and absence of haemoglobin dilution during pregnancy could be considered as indicators of a high-risk pregnancy, regardless of haemoglobin concentration before pregnancy.

## Abbreviations

BMI, body mass index; CI, confidence interval; G/L, germ per litter; Non-SGA, non small for gestational age; OR, odds ratio; SGA, small for gestational age.

## Additional file

Additional file 1: Haemoglobin concentration and the risk of term and preterm stillbirth. (DOC 19 kb)
