# Supplementary material for: Maternal haemoglobin concentrations before and during pregnancy and stillbirth risk: a population-based case-control study
Source: BMC Pregnancy Childbirth. 2016 Jun 3;16:135. doi: 10.1186/s12884-016-0924-x (PMC4893297; doi:10.1186/s12884-016-0924-x)
Supplement: Additional file 1: — Haemoglobin concentration and the risk of term and preterm stillbirth. (DOC 19 kb) [file 12884_2016_924_MOESM1_ESM.doc]

**Additional file:**

| **Additional file 1, Haemoglobin concentration and the risk of term and preterm stillbirth.** | | | | | | | | | | | | |
| --- | --- | --- | --- | --- | --- | --- | --- | --- | --- | --- | --- | --- |
|  | **Preterm stillbirth** | | | | | | **Term stillbirth** | | | | | |
|  | **Cases** | | **Controls** | | **Odds ratio^*^** (95% CI) | | **Cases** | | **Controls** | | **Odds ratio^*^** (95% CI) | |
|  | **n** | **(%)** | **n** | **(%)** |  |  | **n** | **(%)** | **n** | **(%)** |  |  |
| **Haemoglobin concentration before pregnancy** (gr/lit) | | | | | | | | | | | | |
| < 110 | 0 | (0) | 31 | (2.5) | ---- |  | 4 | (3.3) | 30 | (2.6) | 1.45 | (0.46-4.60) |
| 110-120 | 16 | (42.1) | 582 | (47.5) | Reference | | 56 | (46.7) | 548 | (47.6) | Reference | |
| 121-139 | 14 | (36.8) | 360 | (29.4) | 1.29 | (0.57-2.89) | 35 | (29.2) | 339 | (29.4) | 0.91 | (0.57-1.46) |
| ≥140 | 8 | (21.0) | 252 | (21.0) | 0.90 | (0.35-2.34) | 25 | (20.8) | 234 | (20.3) | 0.84 | (0.49-1.43) |
| Continuous | 38 | (100) | 1,225 | (100) | 1.01 | (0.97-1.04) | 120 | (100) | 1,151 | (100) | 1.00 | (0.98-1.01) |
| **Haemoglobin concentration at first trimester** (gr/lit) | | | | | | | | | | | | |
| < 110 | 8 | (6.0) | 181 | (6.5) | 0.83 | (0.37-1.87) | 18 | (5.1) | 171 | (6.6) | 0.85 | (0.49-1.48) |
| 110-120 | 63 | (47.1) | 1,411 | (50.5) | Reference | | 162 | (46.0) | 1,303 | (50.5) | Reference | |
| 121-139 | 34 | (25.4) | 834 | (29.9) | 0.92 | (0.60-1.42) | 160 | (45.4) | 1,056 | (40.9) | 1.20 | (0.93-1.53) |
| ≥140 | 29 | (21,6) | 366 | (13.1) | 1.58 | (0.98-2.55) | 12 | (3.4) | 52 | (2.0) | 1.82 | (0.91-3.65) |
| Continuous | 134 | (100) | 2,792 | (100) | 1.01 | (1.00-1.03) | 352 | (100) | 2,582 | (100) | 1.01 | (1.00-1.02) |
| **Haemoglobin concentration at end of second trimester** (gr/lit) | | | | | | | | | | | | |
| < 110 | 12 | (14.3) | 523 | (21.2) | 0.64 | (0.34-1.21) | 25 | (13.1) | 494 | (21.5) | 0.58 | (0.37-0.91) |
| 110-120 | 55 | (65.5) | 1,618 | (65.6) | Reference | | 125 | (65.4) | 1,506 | (65.4) | Reference | |
| 121-139 | 11 | (13.1) | 268 | (10.9) | 1.15 | (0.59-2.25) | 29 | (15.2) | 246 | (10.7) | 1.35 | (0.88-2.08) |
| ≥140 | 6 | (7.1) | 59 | (2.4) | 2.92 | (1.19-7.18) | 12 | (6.3) | 55 | (2.4) | 2.46 | (1.27-4.75) |
| Continuous | 84 | (100) | 2,468 | (100) | 1.03 | (1.01-1.05) | 191 | (100) | 2,301 | (100) | 1.03 | (1.02-1.04) |
| *Adjusted for maternal age, maternal height, maternal BMI, parity, smoking status, husband’s profession, region and gestational age at haemoglobin measurement. | | | | | | | | | | | | |
